# *In vitro* and *in vivo* biodegradation and biocompatibility assessment of magnesium composites for bone implants

**DOI:** 10.3389/fbioe.2025.1685918

**Published:** 2025-10-03

**Authors:** Samantha Gmitro, Andres Larraza, Pedram Sotoudehbagha, Andres Alayon Mata, Andrew Romero, John Lovejoy, Mehdi Razavi

**Affiliations:** ^1^ Biionix (Bionic Materials, Implants and Interfaces) Cluster, Department of Medicine, University of Central Florida College of Medicine, Orlando, FL, United States; ^2^ Burnett School of Biomedical Sciences, University of Central Florida College of Medicine, Orlando, FL, United States; ^3^ Department of Materials Sciences and Engineering, University of Central Florida, Orlando, FL, United States; ^4^ Department of Orthopaedics, Sports Medicine and Physical Medicine and Rehabilitation, Nemours Children’s Hospital, Orlando, FL, United States; ^5^ Biomedical Engineering Program, Department of Mechanical and Aerospace Engineering, University of Central Florida, Orlando, FL, United States

**Keywords:** biocompatible materials, bone-implant interface, magnesium, nanocomposite, osseointegration

## Abstract

**Introduction:**

With an average of 6.8 million fractures per year in the United States, the current method for surgical intervention involves bioinert materials that do not promote osteogenesis and can have inflammatory reactions that hinder bone healing. Magnesium has emerged in research due to the material’s biodegradability and biocompatibility; however, it has a low corrosion resistance, which can lead to hydrogen gas evolution and tissue necrosis. Therefore, magnesium is typically alloyed with rare earth elements (REEs) to increase corrosion resistance. The main goal of this study involves fabricating a magnesium (Mg)‐based metal matrix nanocomposite (MMNC) containing scandium (Sc) and strontium (Sr) as alloying elements, as well as diopside (CaMgSi2O6) ‐based bioactive glass‐ceramic nanoparticles for reinforcement.

**Methods:**

MMNCs were processed using ultrasonic melt processing and hot rolling to disperse nanoparticles and refine their microstructure. These MMNCs have undergone detailed tests to determine microstructure and degradation properties, followed by *in vitro* and *in vivo* tests to determine the MMNC’s biodegradation and biocompatibility characteristics.

**Results:**

Through cell culture with human bone marrow-derived mesenchymal stem cells (hBM-MSCs) we determined that MMNCs provide *in vitro* cytocompatibility of >80%. Next, MMNC pins were implanted into rat femoral defects and monitored for 3 months post-implantation with the WE43 Mg alloy used as a control. Utilizing *in vivo* and *ex vivo* X-ray imaging and histology of these defects implanted with MMNC or WE43 pins, we found that our composite allows for no or minimal hydrogen gas evolution and fibrotic body response with osteointegration and new bone formation.

**Discussion:**

This allows for an understanding of potential applications of our composite as a biomaterial.

## 1 Introduction

Each year, doctors treat roughly 6.8 million fractures across all age ranges in the United States (Fracture Collaborators et al., 2021). The rate of fracture increases with age due to bone-related diseases such as osteoporosis, with 2 million fractures per year being due to this disease (May 2023). Depending on the severity of the fracture, treatment can involve surgical or non-surgical options to hold the bone together during healing. In the case of surgical intervention, internal fixation devices that utilize metal plates, rods, and screws can be used to stabilize the fracture (Fan et al., 2023). Surgery, alongside other healthcare costs, can total anywhere between $16,000 to $40,000 (Vanderkarr et al., 2023).

Bioinert materials are commonly used for internal fixation devices, such as stainless steel, titanium alloys, and cobalt chromium (CoCr) alloys (Zhou et al., 2023). Stainless steel and CoCr, while having a high corrosion resistance, have an elastic modulus that is significantly higher than that of cortical bone (193 and 220 GPa compared to 10–40 GPa), making the risk of osteolysis and secondary fracture much higher due to stress shielding. These metals also have materials that pose the risk of adverse effects within bone tissue, especially cobalt, which has a toxic effect on osteoclasts and synthesis of type I collagen (Barber et al., 2021). Titanium alloys have a lower elastic modulus (55 GPa) and have a higher biocompatibility *in vivo*; however, there is a lower fatigue resistance, which increases the risk of secondary fractures (Li et al., 2020). Release of wear particles and corrosion ions can lead to aseptic loosening of the implant and osteolysis around the implant site. This event, known as periprosthetic osteolysis (PPOL), occurs due to inflammatory responses typically caused by implant-derived wear particles and debris (Xing et al., 2022). Inflammation caused by this debris leads to osteolysis by increasing the activation of osteoclasts via the RANKL pathway and NF-kB (Torres et al., 2023; Mbalaviele et al., 2017). The activation of osteoclasts causes an increase in bone resorption; when this is accompanied by a decrease in bone formation, osteolysis occurs. Osteolysis can then trigger aseptic loosening of the implant, increasing the risk of secondary fracture and the need for revision surgery (Goodman and Gallo, 2019). According to the 2023 American Joint Replacement Registry (AJRR) Annual Report, over three million arthroplasty surgeries occurred between 2012 and 2022. Of these, 18.37% were hip or knee revision surgeries, where one of the most common reasons for revision was due to infection and aseptic loosening (American Joint Replacement Registry AJRR, 2023).

Magnesium (Mg) and its alloys have been considered promising candidates amongst absorbable metals for use in biomedical applications due to their biodegradability and biocompatibility, as well as having an elastic modulus similar to the natural bone (41–45 GPa versus 10–40 GPa) (Su et al., 2018). These characteristics make the Mg materials promising for orthopedic applications by addressing concerns towards inflammatory response, stress shielding, and the need for revision surgeries (He et al., 2023). Through the activation of the canonical Wnt/β-catenin pathway, Mg^2+^ ions play a role in regulating osteogenesis. When activated, this pathway increases bone formation by facilitating mesenchymal stem cell (MSC) differentiation towards the osteoblast lineage (Hung et al., 2019). However, there are drawbacks to utilizing Mg, such as its high corrosion rate that leads to the evolution of H_2_ gas that can cause tissue necrosis and hinder the bone healing process (Amukarimi and Mozafari, 2021). To combat this, rare earth elements (REEs) are typically alloyed with Mg to increase its corrosion resistance.

In this project, we fabricated a Mg-based metal matrix nanocomposite (MMNC) containing scandium (Sc), Strontium (Sr), and diopside (CaMgSi_2_O_6_)-based bioactive glass-ceramic nanoparticles (BG). Sc is regarded as a biocompatible element for use in implants, and it can enhance Mg’s mechanical and degradation properties by altering the grain structure and forming an oxide layer (Kabir et al., 2023). Sr is found in small amounts in the human body and can promote new bone formation and inhibit the apoptosis of osteoblasts; Sr is also an antibacterial agent that can prevent infection. Strontium ranelate, which is a drug containing Sr, has been used to treat osteoporosis by preventing bone resorption and promoting bone formation. However, it was removed from the market due to adverse side effects (Kolodzlejska et al., 2021; Wang et al., 2023). Bioactive glasses were discovered in the 1960s and have since been used as an implant material due to its ability to bond to living tissues and form an interfacial bone-like hypoxyapatite layer (Fernandes et al., 2018). In our diopside bioactive glass-ceramic nanoparticle, silicon (Si) and calcium (Ca) are included for their roles in bone and cartilage development via activation of type I collagen synthesis and mobilization activity in osteoblasts, as well as Ca’s major presence in the bone structure (Pritchard and Nielsen, 2024; Song, 2017).

**FIGURE 1:**
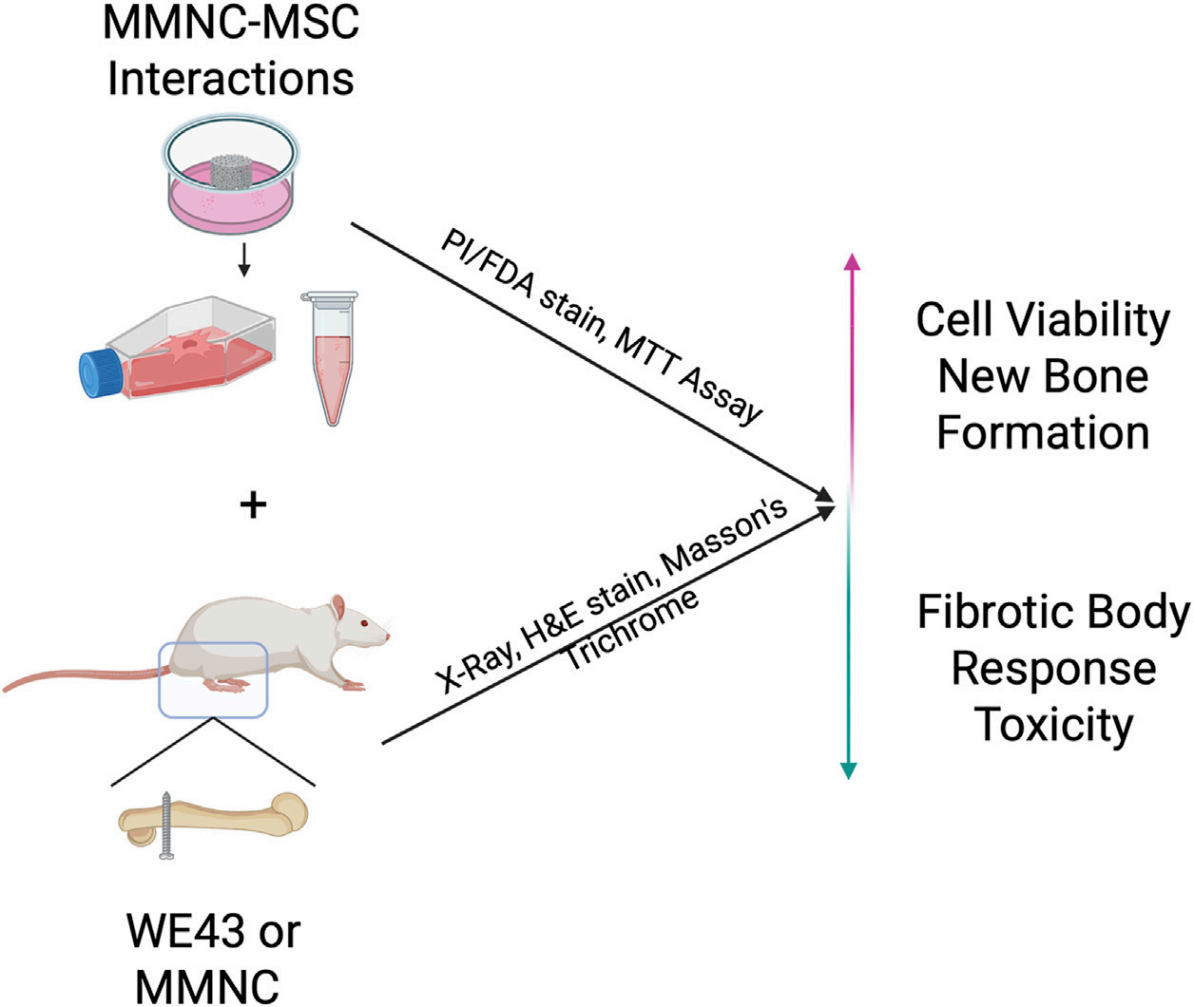
Schematic showing the overall goals of experiments. *In vitro* MMNC-MSC interactions aim to prove cytocompatibility, while *in vivo* implantation aims to show local and systemic biocompatibility, as well as fibrotic body response at the bone-implant interface. Image created with Biorender.com.

In this study, MMNC degradation and cytocompatibility properties were assessed *in vitro*. MMNC-cell interaction was studied using human bone marrow-derived mesenchymal stem cells (hBM-MSCs). Finally, MMNCs were implanted into rat bone defects to evaluate their biodegradation and biocompatibility *in vivo* (Figure 1). The overall goal of this study is to assess the biocompatibility and biodegradation characteristics of our MMNC in comparison to the current FDA-approved Mg alloy, WE43 (Guy et al., 2024).

## 2 Materials and methods

### 2.1 Synthesis of diopside nanoparticles

To generate diopside-based bioactive glass-ceramic nanoparticles (CaMgSi_2_O_6_), a sol-gel method was utilized (Latt et al., 2025). To summarize, 2.1 g of calcium nitrate tetrahydrate (Ca(NO_3_)_2_·H_2_O) (99+%, ACS Reagent; Acros Organics, Morris Plains, NJ, USA) and 2.0 g of magnesium chloride hexahydrate (MgCl_2_·4H_2_O) (99%, Acros Organics, Morris Plains, NJ, United States) were dissolved in 100% ethanol (EtOH) (200-proof, Decon Labs, King of Prussia, PA, United States). When dissolved, the solution was stirred at 250 rpm for 30 min at room temperature. After stirring, 20 mL of tetraethyl orthosilicate (SiC_8_H_20_O_4_) (98%, Acros Organics, Morris Plains, NJ, United States) was dissolved into the solution via magnetic stirring at 450 rpm for 24 h at 80 °C until a gel was formed. To obtain xerogel, this gel was dried in an oven (Model 10–180 Incubator; Quincy Lab, Chicago, IL, United States) at 100 °C for 5 days. This xerogel was milled with a mortar and pestle until a fine powder was generated; and this powder was then calcinated in a furnace (Thermolyne, model FB1315M; Fisher Scientific, Waltham, MA, United States) at 850 °C for 2 h (25 °C/min).

### 2.2 Fabrication and processing of MMNC

To fabricate MMNCs, a metal casting and forming method was utilized (Larraza et al., 2024). In brief, a negative sand mold was utilized with a riser and sprue with a height to reach an approximate laminar flow into the cavity. To begin the casting process, Mg (balance, 96.2 wt%), Sc (3 wt%), and BG (0.5 wt%) were added to a graphite crucible and heated to 930 °C under an argon atmosphere. Sr (0.3 wt%) was added last to prevent oxidation of the melt when casting. This composition was chosen based on our preliminary optimization studies to reduce corrosion, prevent melt oxidation, and maintain biocompatibility. Slag was removed before processing to minimize oxide inclusions. To begin mixing, a graphite rod was used to manually stir for 30 s.

Next, the melt was processed via ultrasound treatment (UST) with a Hielscher UP200st and a ceramic sonotrode. Before insertion of the probe, the melt was heated to 980 °C, and the sonotrode was preheated to prevent solidification of the melt on the probe. The probe was then inserted into the melt and underwent a 1-min treatment consisting of a 6-s on, 4-s off cycle with 1 W power and 50% amplitude of the maximum operating amplitude of 25 µm. When ultrasound processing was completed, the melt was cast and left to cool in the sand mold for two hours.

For heat treatment, samples were placed in quartz tubes covered in aluminum foil to prevent surface oxidation and treated at 400 °C for 6 h. They were then cooled to room temperature in the quartz tubes. To perform hot rolling of the samples, they were first machined to a 6.3 mm diameter before being heat-treated for 1 hour at 400 °C. Samples were then quickly removed from the quartz tubes and rolled to a 6.0 mm diameter. This was then repeated to roll samples to a 5.0 mm diameter.

#### 2.2.1 Assessment of nanoparticle size

The microstructure of UST Rolled MMNC was examined using scanning electron microscopy (SEM) previously (Larraza et al., 2024). Three representative micrographs (n = 3) were selected for quantitative image analysis (in total 344 particles were analyzed). Image processing and particle quantification were performed using ImageJ (NIH, Bethesda, MD, United States). The particle shape and size were quantified using two descriptors: circularity (0–1, where 1 indicates a perfect circle and values closer to 0 represent increasingly elongated shapes) and Equivalent Circular Diameter (ECD), calculated as (Montheil et al., 2022):

$$Circularity=\frac{4\pi \times Area}{{Perimeter}^{2}}$$

$$ECD=2\times \sqrt{\frac{Area}{\pi }}$$

### 2.3 Immersion testing and degradation morphology

Immersion testing was completed in compliance with the ASTM G31 standard (ASTM International, 2021). Hank’s Balanced Salt Solution (HBSS) (Cytiva Life Sciences, Marlborough, MA, United States) was prepared according to manufacturer instructions with deionized water (diH_2_O) and the pH was corrected to 7.4 ± 0.5 via 10 M NaOH and 1 M HCl.

The MMNC discs (5 mm diameter, 4–5 mm thickness) were immersed in HBSS for 1 and 3 days. Once complete, samples were dried in an oven at 100 °C for 15 min and were then stored under vacuum. Corrosion products were removed via immersion in 2 mL of chromic acid (H_2_CrO_4_) and then rinsed with 100% EtOH (200-proof) for 5 min.

After corrosion products were removed, the degradation morphology was assessed using a Keyence VHX700 Microscope, where one ROI was selected on the surface of each sample. Quantification of pit geometry was performed for pit area and diameter using ImageJ. Three-dimensional mapping of corrosion pitting was also performed using the Keyence VHX700 Microscope to determine changes in depth over time.

### 2.4 Human bone marrow-derived mesenchymal stem cell (hBM-MSC) culture

hBM-MSCs (ATCC, Manassas, VA, United States) were cultured in 10 mL of cell culture media containing Dulbecco’s Modified Eagle Medium (DMEM) (Cytiva Life Sciences, Marlborough, MA, United States) with 10% Fetal Bovine Serum (FBS) (Corning, Corning, NY, United States) and 1% Streptomycin/Penicillin (Gibco, Grand Island, NY, United States) in a T-75 flask with a seeding density of 2.1 × 10^6^ cells/mL. Cell culture media was refreshed every 3 days until cells reached a confluency of 8.4 × 10^6^ cells/mL. Once cells reached confluency, they were subcultured into 96-well plates at a seeding density of 0.01 × 10^6^ cells/mL to be used for indirect cell culture with MMNC extract.

#### 2.4.1 Indirect hBM-MSC-MMNC interactions

MMNC discs (n = 6) with dimensions of ∼5 × 2 mm were autoclaved under a dry cycle and then immersed in cell culture media with a ratio of 3 cm^2^/mL in a cell incubator (37 °C, 5% CO_2_) for 72 h. After incubation was complete, cell culture media with extract was collected and diluted to 1.25 cm^2^/mL with cell culture media before being stored at −80 °C until use (International Standard, 2021). Prior to culturing with cells, the media was filtered with a 0.22 μm syringe filter (Fisher Scientific, Waltham, MA, United States) for further sterilization.

To study cell-material interactions, hBM-MSCs were cultured in two 96-well plates at a seeding density of 0.01 × 10^6^ cells/mL with 150 µL of diluted extract media from UST-rolled MMNC immersion or regular cell culture media to serve as a control. Cells were cultured with extract media in a technical triplicate, and one well plate was used for live-dead staining, while the other was used for an MTT assay. Cells were then incubated (37 °C, 5% CO_2_) in a cell incubator for 1 or 7 days before the MTT Assay and Live-Dead staining were performed to determine viability and cytocompatibility.

#### 2.4.2 MTT assay

To determine cytocompatibility, an MTT (3-(4,5-dimethylthiazolyl-2)-2,5-diphenyltetrazolium bromide) Assay was performed with n = 6 samples per group in a technical triplicate per manufacturer’s instructions (Abcam, Cambridge, United Kingdom). Briefly, cells grown in a 96-well plate for 7 days were washed with 1X Phosphate Buffered Saline (PBS), and equal parts of MTT Reagent and DMEM (50 µL) were added to the cells and incubated for 4 h at 37 °C. After 4 h, 150 µL of Dimethyl Sulfoxide (DMSO) was added to each well and incubated in the dark at room temperature for 10 min while on an orbital shaker. The optical density was then read using a microplate reader at 590 nm.

To calculate cytocompatibility, the triplicate readings were averaged, and the background absorbance (MTT assay run with no cells present) was subtracted from these measurements to gain the corrected absorbance. To determine % cell viability, the following calculation was used:

$$\%\;Cell\;Viability=\frac{ODsample}{ODcontrol\;}\times \left(100\right)$$

Where OD_sample_ refers to the corrected absorbance of the sample (cells cultured with MMNC extract media) and OD_control_ refers to the corrected absorbance of the control (cells cultured with regular cell media).

#### 2.4.3 Live-dead staining

To further determine cell viability, Propidium-Iodine/Fluorescein-Diacetate (PI/FDA) staining was performed per manufacturer’s instructions (IBIDI, Fitchburg, Wisconsin, United States) after 1 and 7 days of incubation. Briefly, PI was prepared by adding 2 mg of PI powder to 1 mL of PBS, and FDA was prepared by adding 5 mg of FDA powder to 1 mL of acetone and mixed thoroughly. To begin staining, a staining solution was prepared with 5 mL of DMEM, 8 µL of FDA, and 50 µL of PI. After washing cells with 1X PBS, cells were stained by adding 200 µL of staining solution to each well and incubated in the dark at room temperature for 10 min. The staining solution was then removed and replaced with 1X PBS before analysis with fluorescent microscopy (Leica DMI3000). A GFP filter was used to image live cells, and Texas Red was used to image dead cells. One image was taken per well at a randomly selected ROI where cells were present. Both live and dead cells were counted on ImageJ to discern the % of live cells present at both day 1 and day 7 for both groups.

### 2.5 *In vivo* experiment

Animal surgery was performed according to the Institutional Animal Care and Use Committee (IACUC) at the University of Central Florida (PROTO202300024). Male 14-week-old Sprague Dawley rats from the Charles River Laboratory (CR, Wilmington, MA, United States) weighing between 250 and 300 g were used in this study. Animals were randomly selected with surgeries alternating between utilizing the MMNC and WE43 pins with a total of n = 7 subjects per group. For surgery, subjects were anesthetized with 3% isoflurane inhaled (Covetrus, Portland, ME, United States) with less than 1 L/min of oxygen delivery. Subjects were also given intramuscular injections of 200,000 IU/kg procaine penicillin antibiotic (Pro-Pen-G, Bimeda, Oakbrook Terrace, IL, United States) once prior to surgery and once a day for 3 days post-surgery; as well as 0.65 mg/kg Buprenorphine (ETHIQA XR, Fidelis Animal Health Inc, North Brunswick, NJ, United States) immediately prior to surgery. The subject was placed on a 37 °C heating pad to maintain body temperature, and the skin was prepped using 10% saturated povidone-iodine prep pads (Dynarex, Orangeburg, NY, United States). A 3–4 cm incision was created longitudinal to the proximal femur through both the skin and subcutaneous tissue with a #15-blade scalpel, and the deep fascial layer was also incised longitudinally in line with this incision. Once the bone was exposed using a muscle-splitting approach between the heads of the biceps femoris, a 1.5 mm drill bit was used to create a lateral-to-medial bone defect in the proximal femur. This defect was then filled with a ∼1.45 × 7.50 mm pin made of MMNC (n = 7) or WE43 (n = 7) with a ∼2.5 mm pinhead to prevent the pin from falling out of the defect. Prior to implantation, all pin implants were autoclaved utilizing a dry cycle for sterilization. To close the wound, polyglactin 910, 4–0 vicryl (Ethicon, Raritan, NJ, United States) sutures were used with deep dermal stitches, and this was followed with skin staples utilizing the BD Autoclip™ Wound Closing System. At 24 h post-surgery, recovery was performed under the hood, and each subject was placed in a cage partially on top of a 37 °C tray with a half-opened filter to circulate air. Gel food was placed inside the cages alongside water and normal food. After 14 days, the staples were removed. The subjects were sacrificed 3 months after surgery through euthanasia with CO_2_ inhalation at a flow rate of approximately 12 L/min until respiratory arrest. Major organs (heart, liver, lung, kidney, and spleen), muscle, and femur samples were harvested for histological evaluation.

#### 2.5.1 *In vivo* and *ex vivo* imaging

X-ray imaging was conducted 3 days as well as 2-, 4-, 6-, 8-, 10-, and 12-week post-surgery with an *In-Vitro* Xtreme II Imager (Bruker, Billerica, MA, United States). After sacrificing at 3 months, *ex vivo* imaging was obtained of the femurs when removed from the subject.

#### 2.5.2 Histology

Hematoxylin and Eosin (H&E) staining was performed on the femurs and muscle from both the implantation side and intact side, as well as the heart, kidney, liver, lung, and spleen. Prior to histology, we attempted to manually pull out the implants from the femurs; however, we were unable to due to osseointegration. Femurs were decalcified by being placed in a 5% nitric acid solution for 8 days at 4 °C, and the solution was refreshed on day 4. At the end of decalcification, the implants had dissolved, leaving the remaining defect without impacting the interface. For deparaffinization, samples were dipped 3 times for 5 min each in Clearify clearing agent (StatLab™, McKinney, TX, United States). Slides were then immersed in 100% EtOH twice, 70% EtOH twice, and tap water once for 5 min each to be rehydrated before being stained with Hematoxylin for 2 min (1 min for liver) and Eosin for 1 min with a 3-min wash in between the two stains with tap water. Once staining was complete, samples were dehydrated with 95% EtOH followed by 100% EtOH and then cleared with three rounds of Clearify. Samples were then mounted, and a coverslip was added with a xylene substitute mountant (ThermoFisher, Waltham, MA, United States). Images were taken of staining using an optical microscope (VHX 8000, Keyence, United States) at various magnifications.

Masson’s Trichrome staining was performed on femurs by following manufacturer’s instructions provided through VitroView™ Masson’s Trichrome Stain Kit (United States). Briefly, femur sections were deparaffinized using Clearify as a clearing agent, and rehydration was performed utilizing immersion in a descending gradient with 100% EtOH, 95% EtOH, and 70% EtOH for 1 min each. Samples were then placed in distilled water for 5 min and put through Mordant in Bouin’s Solution at 60 °C for 1 h. After being rinsed in running tap water for 5 min, the staining process continued. This staining process involved application of Wiegert’s working Hematoxylin A and B at a 1:1 ratio for 10 min, rinsing with tap water for 5 min, followed by a rinse with distilled water for 1 min. Samples were then stained with Biebrich Scarlet-Acid Fusion solution for 5 min before being rinsed again in distilled water. They were then immersed in a Phosphomolybdic-Phosphotungstic Acid solution for ten minutes, followed by staining with Aniline Blue for 10 min, and then immersion in an 1% Acetic Acid solution for 2 min. Once complete, samples were dehydrated with 95% and 100% EtOH and cleared with Clearify before being mounted with a coverslip with a xylene substitute mountant (ThermoFisher, Waltham, MA, United States). Images were taken of staining using an optical microscope (VHX 8000, Keyence, United States) at various magnifications and analyzed using ImageJ software to determine new bone formation by measuring aniline blue areas at implant interface. To quantify new bone formation, ImageJ software was utilized with 1-3 ROIs randomly selected per image used. 20 measurements were generated across seven samples. The area of new bone formation, visualized by aniline blue staining, was measured with the area function in the software. Measurements from the three ROIs were then averaged and outliers were excluded when performing analysis.

### 2.6 Statistical analysis

Statistical analysis was performed using GraphPad Prism. A two-way ANOVA was performed for cell viability studies. The Mann-Whitney U test was utilized for corrosion and new bone quantification. Data is considered significant if p ≤ 0.05 and not significant if p > 0.05. Data is presented as mean ± standard error of the mean. For metallography, n = 3 samples were used for imaging. For nanoparticle diameter analysis, three representative micrographs (n = 3) were selected for quantitative image analysis (in total 344 particles were analyzed). Image processing and particle quantification were performed using ImageJ. For *in vitro* experiments, sample sizes of n = 8-9 per group was used for corrosion morphology, and one ROI was selected per sample and imaged to assess pit depth, diameter, and area. For cell viability studies, n = 6 per group was used, and samples were done in technical triplicate. For live-dead staining, an ROI was randomly selected where cells were present to discern live %. For *in vivo* experiments, n = 7 rats per group was used. For histology, images were taken at 40X, 100X, and 200X. Images of implant sites and bone-implant interfaces had images taken at additional magnifications of 80X, 150X, and 500X. The number of images taken at each magnification ranged from n = 1–7 per magnification.

## 3 Results

### 3.1 Corrosion morphology shows pitting degradation mechanism

**FIGURE 2:**
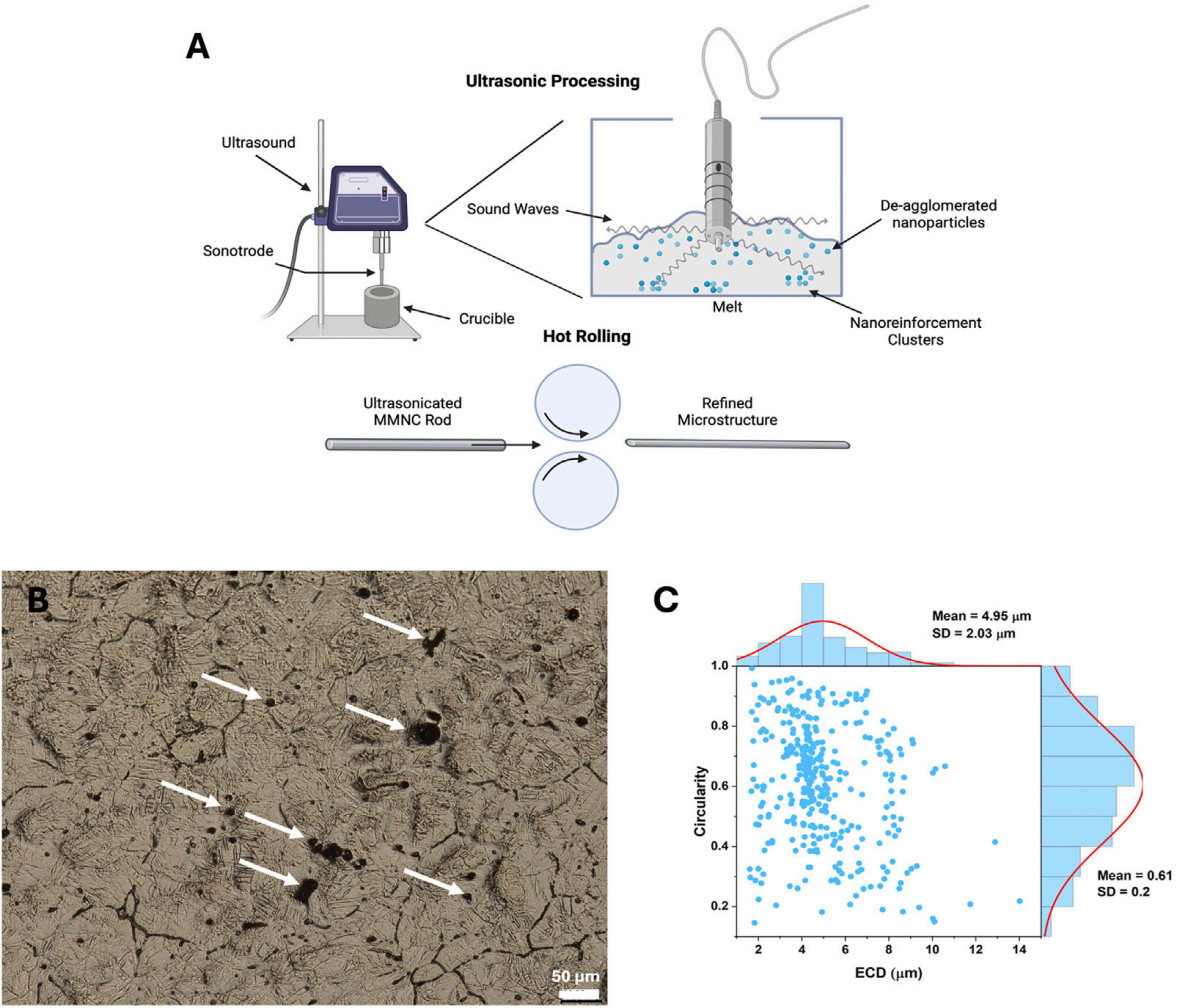
Microstructure assessment of MMNC composites. **(A)** Graphic showing the goal of ultrasonication is to aid in dispersing and de-aggregating nanoparticles in the MMNC melt during fabrication (top) as well as the process of hot rolling (bottom). **(B)** Metallography image of MMNC taken at 400X shows grain structure and dispersion of nanoparticles. **(C)** Circularity versus Equivalent Circular Diameter (ECD) for UST Rolled MMNC quantified from SEM images. Circularity (0–1) indicates particle roundness, where 1.0 represents a perfect circle and values closer to 0.0 indicate elongated shapes. A normal size distribution curve was fitted to the data. Arrows show location of nanoparticle clusters. Scale bar is 50 μm. **(A)** was created using Biorender.com.

Samples were treated with ultrasonication and hot rolling to aid in dispersing nanoparticles (Figure 2A). Before immersion, images were taken of sample surfaces to show microstructure and nanoparticle dispersion (Figure 2B). In our previous work, we investigated the microstructure of rolled UST MMNC, which exhibited smaller, more spherical particles with a uniform distribution (Larraza et al., 2024). EDS analysis further confirmed the incorporation of BG, showing the presence of Si and Ca. In the present study, particle shape and size were quantified using two descriptors: circularity (0–1, where 1 indicates a perfect circle and values closer to 0 represent increasingly oval-like shapes) and ECD (Figure 2C). The mean values obtained were 0.61 ± 0.20 for Circularity and 4.95 ± 2.03 μm for ECD, with the relationship depicted in Figure 2C.

**FIGURE 3:**
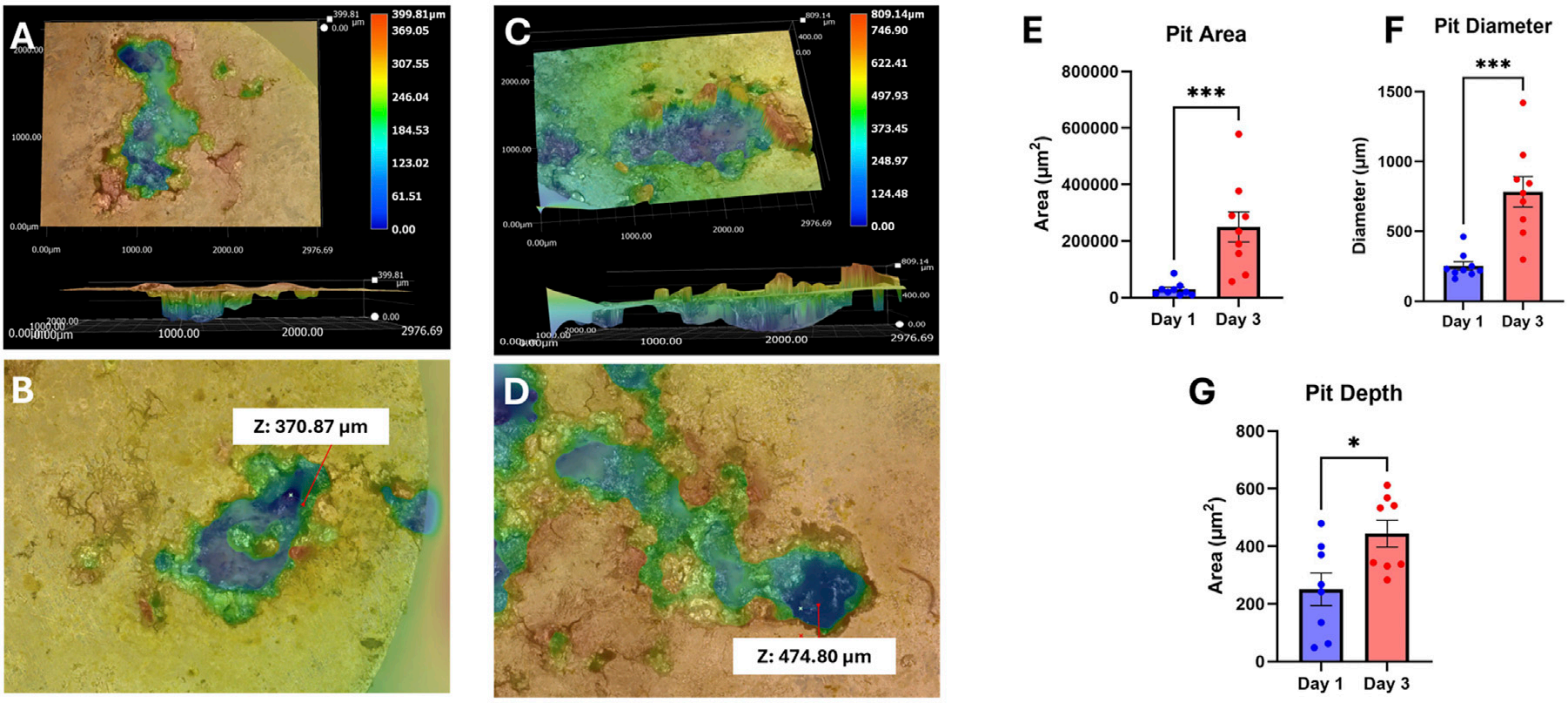
3D corrosion morphology images of UST MMNCs after 1 day **(A,B)** and 3 days **(C,D)** of immersion in HBSS, followed by corrosion product removal using chromic acid, showing pit formation and growth over time. The surface area, diameter, and depth of pits increased from day 1 to day 3. Z refers to pit depth. **(E–G)** Corrosion analysis of area, diameter, and depth. Pit area **(E)**, diameter **(F)**, and depth **(G)** increased over the 3 days of immersion. Statistical significance: * p-value <0.05, *** p-value <0.001. n = 8-9 per group. Mann-Whitney test.

Images were taken again after immersion testing to visualize the corrosion mechanism of our UST MMNCs (Figures 3A–D). We found that our samples underwent pitting corrosion, with the pit diameter increasing between days 1 and 3 from 253 ± 30.09 μm to 783 ± 108.7 μm (p-value = 0.0002) and the area also increased from 28,801 ± 8,012 μm^2^ to 249,851 ± 53,450 μm^2^ (p-value = 0.0002). (Figures 3E,F). Pit depth also increased between days 1 and 3 from 250.8 ± 56.57 μm to 444.2 ± 46.42 μm (p-value = 0.0379) (Figure 3G). Overall, this reflects that our samples were able to undergo degradation with a pit corrosion mechanism *in vitro*, with significant increases in pit size and depth over 3 days.

### 3.2 MMNCs show biocompatibility *in vitro*

**FIGURE 4:**
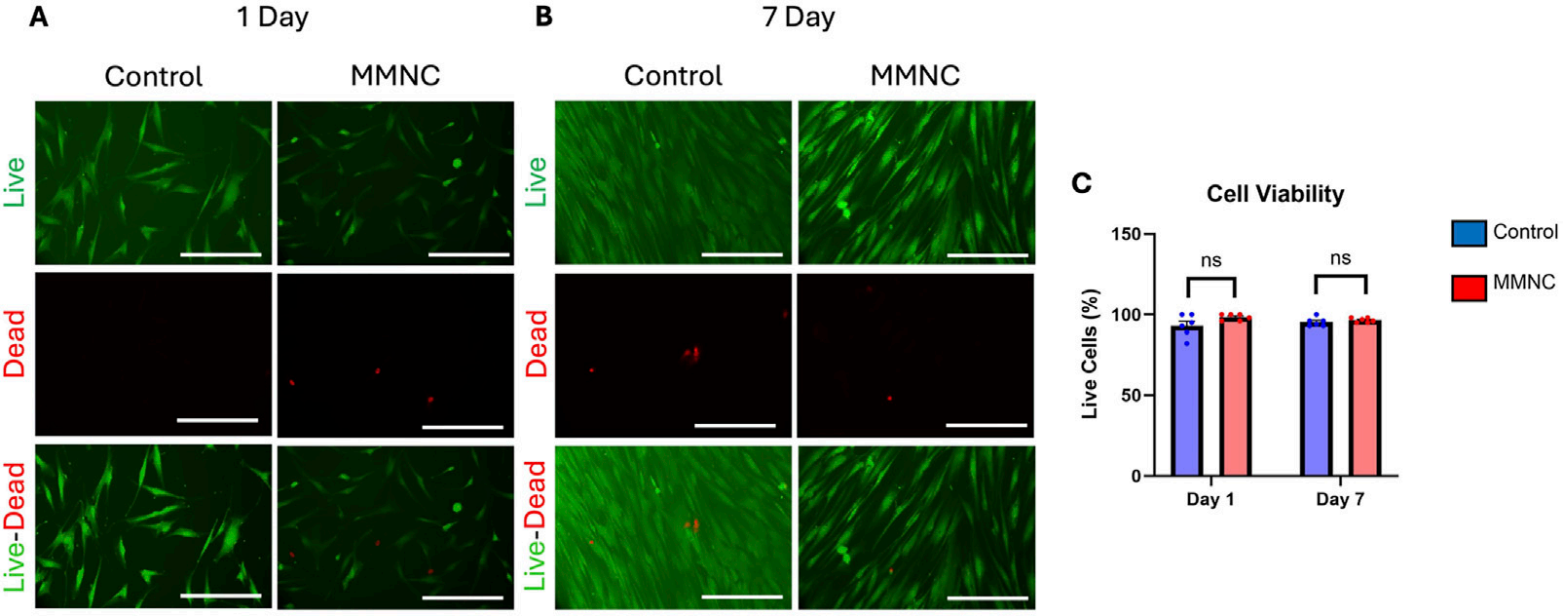
Cell viability of MSCs treated with or without MMNC extract. PI-FDA Live/Dead staining was performed after 1 day **(A)** and 7 days **(B)** to show live cells (green, top), dead cells (red, middle), and a merged image (bottom). **(C)** Quantification and comparison of cell viability through live-dead assay. Statistical significance: ns = not significant, p-value >0.05. n = 6 per group. Two-way ANOVA. Scale bar represents 100 μm.

To investigate whether our MMNC meets the biocompatibility standards set by ISO 10993 *in vitro*, we performed PI-FDA Live/Dead staining and MTT assays on hBM-MSCs cultured indirectly with extract collected from immersing MMNCs in cell culture media. After incubation for either 1 or 7 days, we found through staining that cell viability remained high after 7 days of incubation (Figures 4A,B). In treated cells, we found a live cell % of 97% ± 1%; this is comparable to our control cells, where there was a live cell % of 95% ± 1% after 7 days (Figure 4C). While there was not a significant change in cell viability, treatment with MMNC extract was not cytotoxic to cells over 7 days.

We also performed MTT assays to further confirm *in vitro* cytocompatibility. When performing MTT assays on both treated and untreated cells after 7 days, we found cells treated with the MMNC extract had a percent cell viability of 89.55% ± 2% when compared to the control.

### 3.3 X-ray imaging shows lack of hydrogen gas pocket formation

**FIGURE 5:**
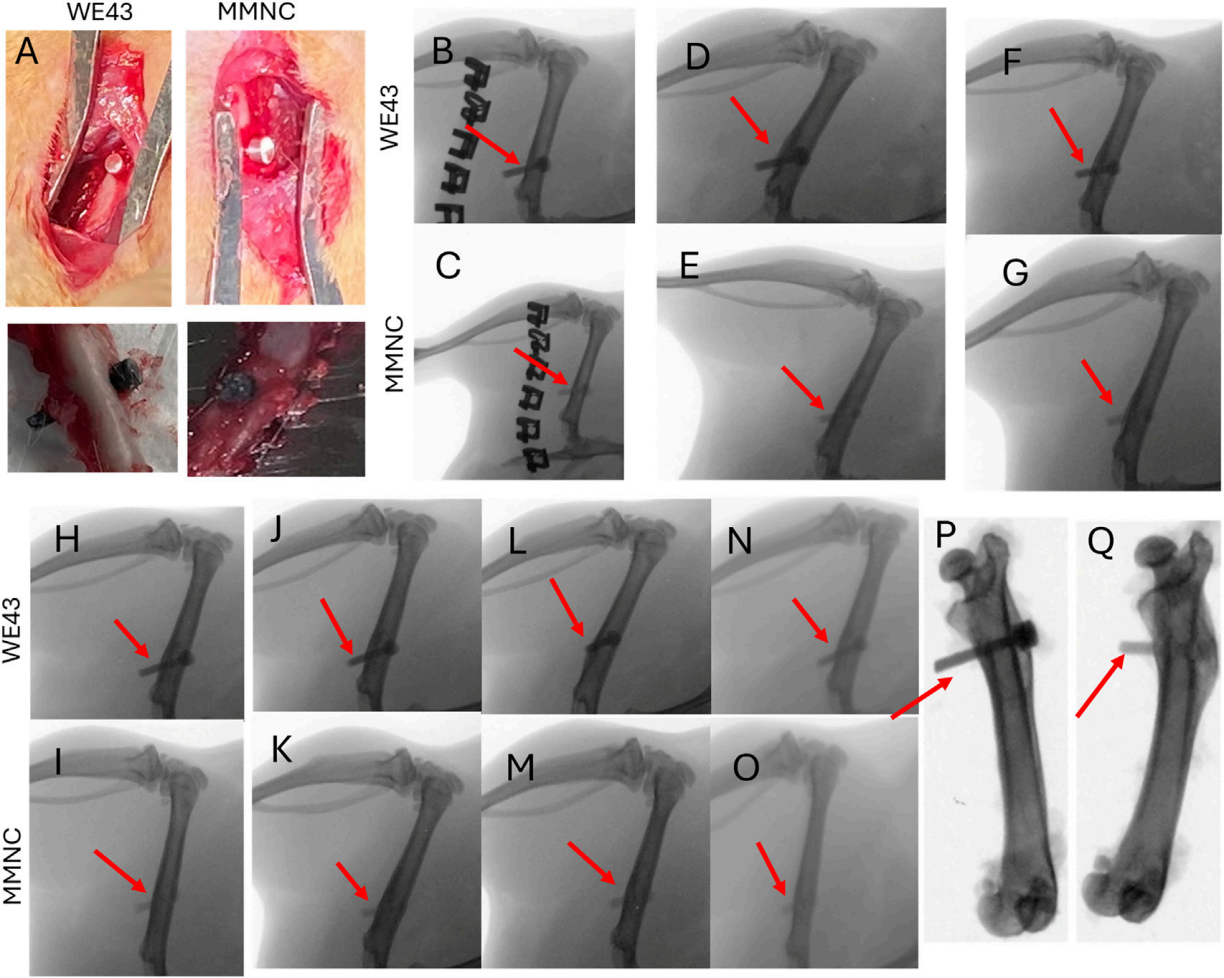
Surgical and X-Ray images of implantation of pins into rat femurs. **(A)** Implantation (top) and explantation (bottom) of WE43 and MMNC pins. X-Ray images were taken at day 3 **(B,C)**, week 2 **(D,E)**, week 4 **(F,G)**, week 6 **(H,I)**, week 8 **(J,K)**, week 10 **(L,M)**, and week 12 **(N,O)** for WE43 (top) and MMNC (bottom) implantation sites. **(P,Q)** *Ex vivo* X-ray images of WE43 **(P)** and MMNC **(Q)**. Red arrows indicate the location of the implant. n = 7 rats per group.

Pins composed of either MMNC or WE43 were implanted into the rat femur (Figure 5A, top) and then explanted after 3 months (Figure 5A, bottom). X-ray images were taken of rat femurs on day 3 post-implantation, followed by every 2 weeks until 3 months (12 weeks) had passed. Through X-ray imaging, we were able to determine that neither our MMNC nor the WE43 implant produced any detectable hydrogen gas formation in the area surrounding the implants, as there was no discoloration surrounding the implant site (Figures 5B–O). In addition, we were able to conclude that both MMNC and WE43 implants maintained their stability and structural integrity over the course of the study through *ex vivo* X-ray imaging. These images showed that both MMNC and WE43 implants were still present in the femur, which allowed us to conclude that these implants did not shift locations due to implant loosening throughout the 12 weeks (Figures 5P,Q). This also allowed us to note that both MMNC and WE43 implants had become well integrated into the surrounding bone tissue, as we were unable to pull out the implants from the femur manually at the time of sample collection because of their high osteointegration.

### 3.4 *In vivo* study reflects biocompatibility of implants

**FIGURE 6:**
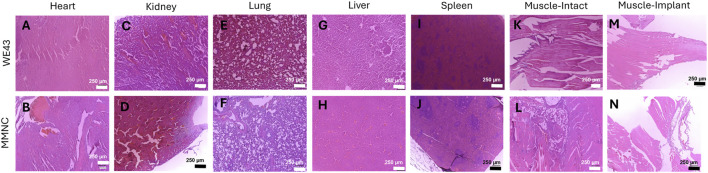
Hematoxylin and Eosin staining on vital organs shows systemic biocompatibility. Histology was performed on sections of the heart **(A,B)**, kidney **(C,D)**, lung **(E,F)**, liver **(G,H)**, and spleen **(I,J)**. **(K–N)** Muscle sections were taken adjacent to the intact femur **(K, L)** and implant femur **(M, N)** for both WE43 (top) and MMNC (bottom). Images were taken at 100X with a scale bar of 250 μm. n = 7 rats per group, 3 slides were stained per organ, images taken at 40X, 100X, and 200X, with representative images at 100X selected.

H&E staining was carried out on the following vital organs to aid in determining *in vivo* biocompatibility: heart, liver, kidney, spleen, lung, and muscle. We found that both MMNC and WE43 tissues showed no abnormalities in tissue structure and little to no presence of an inflammatory response (Figures 6A–J). When comparing muscle sections taken from locations near the intact femur versus the implant site, we found both showed similar tissue structure (Figures 6K–N). This shows systemic biocompatibility *in vivo* due to lack of tissue structure changes caused by systemic toxicity throughout the course of this 3-month study.

**FIGURE 7:**
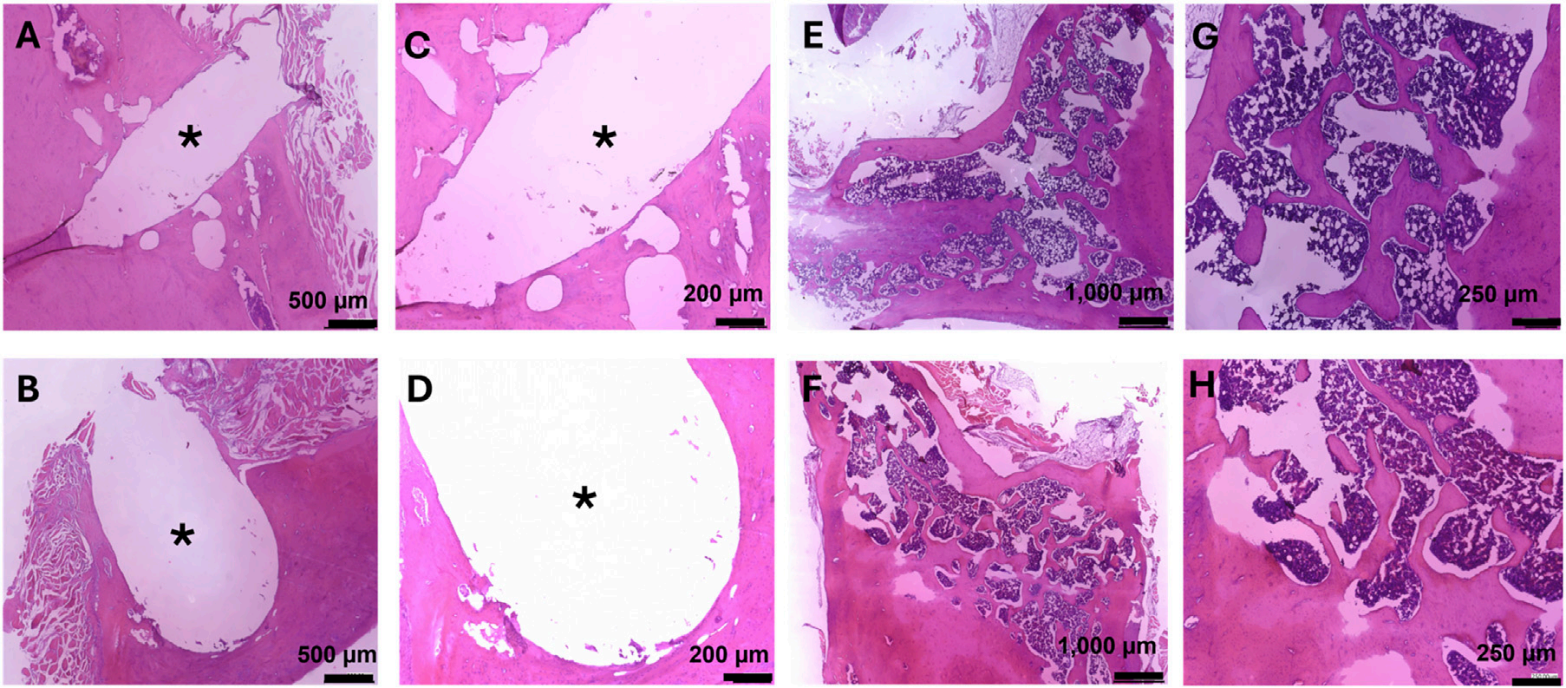
Hematoxylin and Eosin staining on femurs for bone growth, fibrotic body response, and local biocompatibility. Images at the bone-implant interface were taken at 80X **(A,B)** and 150X **(C,D)**. **(A,C)** Bone implant interface of the femur implanted with MMNC. **(B,D)** Bone implant interface of the femur implanted with WE43. **(E–H)** Microstructure of femurs showing cortical and trabecular bone, as well as local biocompatibility for MMNC **(E,G)** and WE43 **(F,H)** taken at 40X **(E,F)** and 100X **(G,H)**. Scale bars are as follows: 40X = 1,000 μm, 80X = 500 μm, 100X = 250 μm, 150X = 200 μm. n = 7 rats per group. 1-3 slides were stained per femur. Images of the femur were taken at 40X, and 100X. Images of the implant site were additionally taken at 80X and 150X. * indicates the location of the implant.

In addition, H&E staining on femurs also showed no tissue abnormalities (Figure 7). The microstructure of the bone was normal, with both organized cortical and trabecular bone found (Figures 7E–H). At the site of implantation, there was little to no detectable fibrotic body response found through the lack of collagen deposits at the implant interface (Figures 7A–D). Overall, this supports the local biocompatibility of the composite during the course of this 3-month study.

### 3.5 New bone formation at site of implantation

**FIGURE 8:**
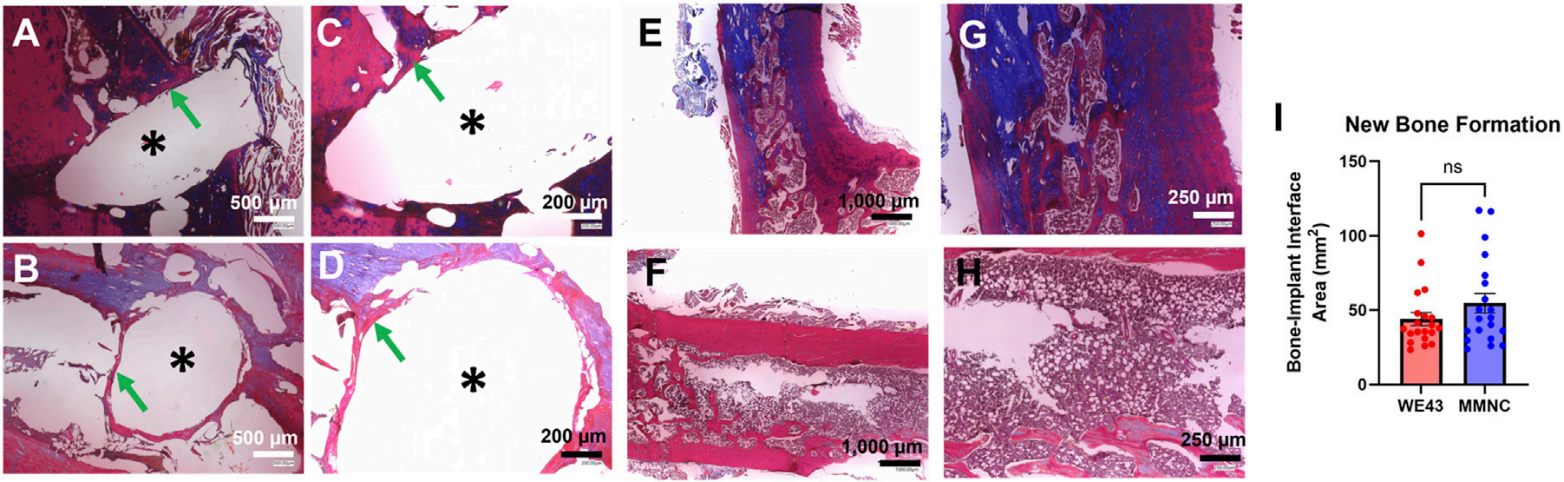
Masson’s Trichrome staining on femurs to detect new bone formation and fibrotic body response. Images at the bone-implant interface were taken at 80X **(A,B)** and 150X **(C,D)**. **(A,C)** Bone implant interface of the femur implanted with MMNC. **(B,D)** Bone implant interface of femur implanted with WE43. **(E–H)** Microstructure of MMNC **(E,G)** and WE43 **(F,H)** femurs showing cortical and trabecular bone at 40X **(E,F)** and 100X **(G,H)**. **(I)** Quantification of new bone formation at the bone-implant interface using ImageJ. Scale bars are as follows: 40X = 1,000 μm, 80X = 500 μm, 100X = 250 μm, 150X = 200 μm. n = 7 rats per group, for quantification n = 20 measurements per group with n = 1–3 measurements taken per sample. Images of the femur were taken at 40X, 100X, and 200X. Images of the implant site were additionally taken at 80X, 150X, and 500X. * indicates the location of the implant. Statistical Significance: ns = not significant, p-value >0.05. Mann-Whitney Test.

Masson’s Trichrome staining was utilized on femurs to study new bone formation and fibrotic body response at the site of the defect, as well as to study bone microstructure. We found similar microstructure of the cortex and trabecular bone between femurs that underwent implantation compared to the intact femurs for both implant groups (Figures 8E–H). In addition, we were able to identify new bone formation at the site of the implant and found 54.65 ± 6.591 mm^2^ of new bone formation in MMNC implant sites compared to 43.97 ± 4.391 mm^2^ at the site of WE43 implants (Figures 8A–D,I). Lastly, we detected no to minimal fibrotic body responses at the implant interface of some implants in both MMNC and WE43 groups (Figures 8A–D). Overall, these results allowed us to conclude that our MMNC does allow for osteopromotion with no or minimal fibrotic body response (FBR).

## 4 Discussion

In summary, this study aimed to determine the biodegradability and biocompatibility properties of our MMNC composed of magnesium, strontium, scandium, and bioactive glass-ceramic nanoparticles compared to the current FDA-approved magnesium alloy, WE43. Because there are roughly 6.8 million fractures treated per year in the United States (Fracture Collaborators et al., 2021), with the occurrence increasing as individuals age, it is important to develop treatment options that not only prevent the need for secondary surgeries but also contain bioactive materials that aid in the bone healing process. Therefore, we created a bioactive composite containing materials shown to play a role in bone metabolism and homeostasis while also being biodegradable.

Mg has emerged in research fields for its use as an absorbable material in biomedical applications because of its biocompatibility and biodegradability through “inflammaging” and the canonical Wnt-β-catenin pathway, as Mg^2+^ ions play a role in in aging and osteogenesis (Hung et al., 2019). Since Mg has a low corrosion resistance, other metals are typically alloyed with it to increase this resistance and add additional properties to the alloy. Here, we chose to add Sc, Sr, and a diopside (CaMgSi_2_O_6_)-based bioactive glass-ceramic nanoparticle containing Ca and Si to create our MMNC. Sc, while not naturally present in the human body, has been shown to be biocompatible and increases the mechanical and corrosion properties through alteration of the grain structure and oxide layer formation (Kabir et al., 2023). Recent studies regarding Sc^3+^ ions have shown promotion of osteogenesis through the Wnt/β-catenin pathway and enhancement of angiogenesis (Wang et al., 2025). Sr, a trace element in the human body, has dual action capabilities through promoting new bone formation and inhibiting bone resorption through various cell signaling pathways, including those that increase expression of osteogenesis-related genes and inhibiting apoptosis of osteoblasts (Kolodzlejska et al., 2021). This capability has been researched as a therapeutic application in osteoporosis and has been utilized in the drug Strontium Ranelate. Lastly, Sr presents antibacterial capabilities aiding in the prevention of infection (Ren et al., 2022). Ca and Si, found in our bioglass, are also included for their roles in bone and cartilage development. These ions are able to activate collagen type I synthesis and mobilization of osteoblasts in the bone structure (Pritchard and Nielsen, 2024; Song, 2017). The specific mechanisms of Si’s actions in bone regeneration are still being understood, but proposed actions include vasculogenesis, synthesis and stabilization of collagen within the extracellular matrix (ECM), and may make the bone matrix more calcifiable (Arora and Arora, 2017). Ca is highly integral to bone structure, with 99% of calcium in the body being stored in the bone as hydroxyapatite (Ca_10_(PO_4_)_6_(OH)_2_), providing the skeleton with its strength as well as a storage center for calcium that can be released into the bloodstream when needed (Yu and Sharma, 2023). Overall, the ions we have chosen to include in our MMNC provide aid in the bone remodeling system through various mechanisms, including the Wnt/β-catenin pathway (Hu et al., 2024).

Although the biocompatibility of these elements has been studied previously by researchers in various studies (Dutta et al., 2022; Taha et al., 2020), the ion release kinetics in combination provides a new avenue of research. Our biodegradable composite, which provides a multi-ionic delivery system, has not been studied previously and may provide enhanced bone healing through *in vivo* models. This composite meets the requirements set by ISO 10993, which states that to be considered biocompatible, there must be at least 80% cell viability (International Standard, 2018). In addition, through *in vivo* modeling, we were able to see no or minimal H_2_ gas bubble formation at the site of the implant through 3 months, as well as high levels of osteointegration, no or minimal FBR, and new bone formation. We believe this is due to the bone regenerative effects of the bioactive ions released from our MMNC composite on bone due to MMNC biodegradability. Including biocompatible components that are found in the human body allows for the reduction of FBR and other pro-inflammatory responses (Amara et al., 2023). In addition, these ions can play roles in various cell signaling pathways that can aid in the increased expression of osteogenic genes, allowing for new bone formation (Bai et al., 2023). Further studies will allow us to utilize osteogenic and inflammatory markers to gain a better understanding of this osteogenesis and any osteoimmunomodulation, as well as confirm the release of these ions from our composite.

We have shown in previous studies that our nanoparticles are dispersed throughout our composite (Larraza et al., 2024); and further analysis in this study proves that UST rolling promotes the formation of smaller, more spherical particles with a mean circularity of 0.61 ± 0.20 and an average ECD of 4.95 ± 2.03 μm. Such morphology is expected to enhance particle dispersion within the matrix, contributing to improved mechanical integrity and potentially better corrosion resistance. The findings are consistent with our earlier work, further confirming the effectiveness of UST rolling in refining MMNC microstructures (Larraza et al., 2024). In these studies, we also performed in-depth analyses comparing the corrosion of WE43 and UST rolled MMNCs alongside other processing techniques, finding that our UST rolled MMNCs have a lower corrosion rate and similar corrosion current density as WE43 (Larraza et al., 2024). We also discovered our composite has statistically consistent corrosion. Due to this, we wanted to further analyze the corrosion morphology of our composite to visualize the specific corrosion mechanism that is occurring at the surface. We have also shown that our MMNC follows a pitting corrosion mechanism through the significant increase in pit depth and diameter. This could be due to microgalvanic corrosion and interfacial voids (Kayani et al., 2024). With further optimization of the microstructure and composition of our material, we can potentially shift this mechanism to uniform corrosion and decrease the amount of pitting that occurs. This can be done by refining the microstructure to improve reinforcement dispersion as well as the better de-agglomeration of nanoparticles. Since pitting corrosion can lead to a pit-to-crack transition that can cause implant loosening and failure, decreasing the amount of pitting corrosion and shifting the mechanism to uniform corrosion is important in ensuring the success of implantation and preventing the need for additional surgeries (Wu et al., 2021; Abdallah et al., 2020; Van Gaalen et al., 2022). This may also aid in promoting biocompatibility and minimizing the possibility of toxic responses within the body.

Although our composite has shown only marginal benefits over the current FDA-approved WE43, we believe that with further optimization we can continue to increase the advantages over materials that are currently used. Through additional studies utilizing additional timepoints and various models (i.e., an osteoporotic model) we can show that our composite holds an increased ability in regeneration and cytocompatibility that goes beyond that of WE43. In addition, the materials present in our composite are all shown to play various roles in osteogenesis and bone healing. With more optimization of our grain structure and nano reinforcement dispersion, we hope to increase the advantages of our composite.

Systemic biocompatibility is another important factor that these materials must be able to deliver upon when interacting with living tissues. To provide evidence that our material supports systemic biocompatibility, we performed histology on various vital organs. Through this, we found no detectable tissue abnormalities in these organs, giving support that the ions released by our MMNC do not provide any off-target toxic effects to other tissues. Further immunohistochemical studies can be performed to further support that there is no inflammatory response occurring at the vital organs.

While we have shown our composite’s ability to promote bone healing in healthy models, this composite may also be applicable towards enhancing the bone repair and regeneration that is imbalanced in osteoporotic models. Through promoting osteoblast formation and osteogenesis, our composite may be able to play a role in improving the deficit of new bone formation compared to bone resorption. Further studies utilizing aged mesenchymal stem cells and osteoporotic rat models will allow us to further pursue this avenue of clinical application.

## 5 Conclusion

Through this study, we aimed to determine the biodegradability and biocompatibility of our MMNC through both *in vitro* and *in vivo* methods. *In vitro* studies involving indirect culture of MMNC extract with mesenchymal stem cells revealed that our composite provides cytocompatibility, as cell viability over the course of 7 days was 89.55%. *In vivo* studies were performed with rat models with a femur defect filled with a pin implant composed of our MMNC or the current FDA-approved alloy WE43. Studies involving X-ray imaging and histology showed that our composite, alongside WE43, provided high osteointegration over 3 months with new bone formation and little to no fibrotic body response and hydrogen gas evolution. Future studies can further confirm osteogenesis and immune responses, as well as study *in vitro* changes in expression of osteogenic and inflammatory genes. In total, we determined that our MMNC allows for both *in vitro* and *in vivo* biodegradability and biocompatibility.

## Data Availability

The raw data supporting the conclusions of this article will be made available by the authors, without undue reservation.
